# The influence of digital competences, self-organization, and independent learning abilities on students’ acceptance of digital learning

**DOI:** 10.1186/s41239-022-00350-w

**Published:** 2022-08-26

**Authors:** Laura Scheel, Gergana Vladova, André Ullrich

**Affiliations:** 1 Chair of Business Informatics, Processes and Systems, University of Potsdam, August-Bebel-Str. 89, 14482 Potsdam, Germany; 2 Research Group Education and Advanced Training in the Digital Society, Weizenbaum Institute for the Networked Society, Hardenbergstraße 32, 10623 Berlin, Germany

**Keywords:** Digital learning, Technology acceptance model, Digital competences, Self-organization, Independent learning, Higher education

## Abstract

Despite digital learning disrupting traditional learning concepts and activities in higher education, for the successful integration of digital learning, the use and acceptance of the students are essential. This acceptance depends in turn on students’ characteristics and dispositions, among other factors. In our study, we investigated the influence of digital competences, self-organization, and independent learning abilities on students’ acceptance of digital learning and the influence of their acceptance on the resistance to the change from face-to-face to digital learning. To do so, we surveyed 350 students and analyzed the impact of the different dispositions using ordinary least squares regression analysis. We could confirm a significant positive influence of all the tested dispositions on the acceptance of digital learning. With the results, we can contribute to further investigating the underlying factors that can lead to more positive student perceptions of digital learning and build a foundation for future strategies of implementing digital learning into higher education successfully.

## Introduction

The use of information and communication technology (ICT) in education is disrupting traditional learning concepts and activities, especially in higher education (Allen & Seaman, 2013; Händel et al., 2020). However, the satisfaction and acceptance of digital learning, which is essential for its effectiveness and success (Dabbagh, 2007; Kreidl, 2011), is perceived differently by students, for example, based on the study subject (Vladova et al., 2021), or student characteristics and dispositions (Stokes, 2001).

Compared to face-to-face, the requirements change in digital learning, and certain dispositions of students become even more significant. These dispositions particularly include digital competences such as accessing, analyzing, and integrating information from various digital sources and evaluating this information and knowledge effectively and ethically (Shopova, 2014). Moreover, self-organization abilities are key, such as integrating digital learning activities into everyday life and managing the time for these activities proactively and effectively (Bernard et al., 2004; Hill & Wouters, 2010). Finally, independent learning abilities are vital, which include the responsibility for learning, self-confidence in mastering new activities, openness to novel experiences, and intrinsic motivation (Macaskill & Taylor, 2010; Warschauer, 2007).

These dispositions have a significant impact on the digital learning process and the use of the respective technologies, which is mainly characterized by students having to learn independently without the direct support of a teacher or other students. Nevertheless, students still appreciate the opportunities to learn and discuss with their peers in person (Radha et al., 2020) as well as the personal contact with teachers. Students were reported to wish for classroom teaching during the COVID-19 pandemic (Giovannella, 2021). Students see face-to-face learning as more real and fear that digital learning would be more difficult or that instructors would not be able to adequately support them in their learning processes (Akcil & Bastas, 2020; Bessette, 2020). These student perceptions influence their attitude toward digital learning, leading to increased uncertainty and doubts about the success of transitioning from face-to-face to digital learning (Akcil & Bastas, 2020).

The literature on digital learning mainly focuses on the learning content and format and less on the learners and psychological factors and behavior (Noskova et al., 2021). Moreover, it is overlooked that digital learning is primarily a cognitive process (Chitkushev et al., 2014). Therefore, to use the respective technologies successfully, it is vital to consider the individual dispositions of students in accepting digital learning in the long term (Mosca et al., 2019). Thus, the goal of this paper is to investigate the influence of the students’ dispositions on digital competences, self-organization, and independent learning abilities. We address this goal with the following question:

### How do digital competences, self-organization, and independent learning abilities influence students’ acceptance of digital learning?

In particular, we apply well-known constructs of digital competences (cf. Rubach & Lazarides, 2021), self-organization abilities (cf. Klein et al., 2021), and independent learning abilities (cf. Macaskill & Taylor, 2010) to investigate their influence on students’ acceptance of digital learning. In addition, we examine the influence of students’ acceptance on the resistance to the change from face-to-face to digital learning (cf. Kim & Kankanhalli, 2009). For the empirical investigation of the acceptance, the technology acceptance model (TAM) by Davis (1989) is adapted, which is widely used in digital learning research (Šumak et al., 2011). As a result, we gain deeper knowledge about possible reasons for the aversion to digital learning. Consequently, recommendations for action from teachers and decision-makers and strategies for digital learning success can be derived and possible challenges of the students addressed.

We first present a theoretical basis for the used constructs. Then, we formulate a research model comprising hypotheses that relate different dispositions to the TAM, empirically testing these relationships using ordinary least squares (OLS) regression analysis and discussing the results. The paper concludes with a summary offering theoretical and practical implications, limitations, and implications for future research.

## Theoretical background

### Digital learning and students’ dispositions

Diverse advantages increase learning efficiency and improve students’ academic performance (Lin et al., 2017; Mothibi, 2015). These advantages include the ability to perform activities independent of time, place, and physical interaction with instructors (Anthonysamy et al., 2020; Coker, 2020; Kümmel et al., 2020) as well as the simplified access to training, interaction, and communication (Kümmel et al., 2020; Sangrà et al., 2012). Additionally, the rapid development and increasing quality of digital technologies for learning, particularly in higher education, represent another reason why digital learning is one of the key components of education in the twenty-first century (Mothibi, 2015). Such technologies include technologies to access and study learning materials, to enable learning collaboration and communication, to assess learners and learning outcomes, to enable a learning-by-doing approach through construction and programming, and to develop digital and multimedia literacy (Bergdahl et al., 2018). Despite the advantages supporting the future viability of digital learning, it differs from face-to-face learning in placing some cognitive requirements on students that represent challenges to certain student dispositions, including digital competences (Shopova, 2014), self-organization abilities (Hill & Wouters, 2010), and independent learning abilities (Macaskill & Taylor, 2010).

Different forms of competences are currently demanded to participate effectively in everyday life, work, and as citizens in a society that considers knowledge as a primary asset (Ananiadou & Claro, 2009; Shavelson, 2013). An essential part of these twenty-first-century competences is digital competences (Ferrari et al., 2012), which are described in various frameworks (Almerich et al., 2020; Coker, 2020).

Rubach and Lazarides (2021) developed an instrument to measure the competences based on, for example, the Digital Competence Framework for Citizens (DigComp) by the European Commission (Carretero et al., 2017), the competences from the German policy framework regarding education in digital spaces (Kultusministerkonferenz, 2016), and the ICT competence dimensions by Siddiq et al. (2016): (1) information and data literacy, (2) communication and collaboration, (3) digital content creation, (4) safety and security, (5) problem-solving, and (6) analyzing and reflecting. (1) Information and data literacy includes searching, filtering, and evaluating data and information, which also represent important activities during digital learning, for instance, when researching information for an assignment or having to critically evaluate the various sources. (2) Communication and collaboration include interacting, sharing, and collaborating through digital technologies and citing information from others correctly. These competences are important for digital learning since collaborating and communicating are mainly possible digitally. (3) Digital content creation involves developing and integrating different digital content, but also copyrights and licenses, which play a key role for students who must share content. (4) Safety and security cover the competences of protecting devices, personal data, well-being, and the environment. During digital learning, students rely on digital technology and the safe handling of data and information. (5) Problem-solving includes solving technical problems and identifying needs and technological responses, which are particularly important during digital learning because students must solve problems mainly on their own. Lastly, (6) analyzing and reflecting refers to evaluating, analyzing, and reflecting on the technologies used. This classification of digital competences will be used for this paper as it includes competences relevant for digital learning (cf. Abrosimova, 2020; Dabbagh, 2007; Shopova, 2014), including managing new digital technologies and having the cognitive skills to use them correctly (Noskova et al., 2021).

Requirements for the success in digital learning are, moreover, self-organization abilities, particularly in higher education (Hill & Wouters, 2010; Yakovleva et al., 2020). These abilities demonstrate how motivationally, metacognitively, and behaviorally engaged students are in their learning process. These factors include the proactive role and self-determined strategies students use to gain academic success and reach certain goals in the learning process (Klein et al., 2021; Zimmerman, 1989). Self-organization contains various self-processes, such as planning and self-control (Kostromina, 2013). Despite self-organization being essential to the general learning process, its relevance increases as it includes the ability to integrate digital learning activities into everyday life (Hill & Wouters, 2010), and manage the time for these activities proactively and effectively (Bernard et al., 2004; Dabbagh, 2007). During digital learning, there are no scheduled classes, and established study routines are disturbed (Costa et al., 2018). All of these facets show that self-organization abilities are related to higher success in academic activities of students in general (Claro & Loeb, 2019; Klein et al., 2021; Kostromina, 2013), including digital learning (Anthonysamy et al., 2020).

In addition to self-organization, digital learning also involves independent learning in the way that the teacher does not have a central role in the learning process (Warschauer, 2007). According to Moore (1973), “independent learning and teaching is an educational system in which the learner is autonomous, and separated from his teacher by space and time, so that communication is by print, electronic, or other non-human medium” (p. 663). In independent learning, there is also a shift of power in the relationship between teacher and learner in favor of the learner (Chene, 1983). Additionally, the student must try to solve problems independently (Abrosimova, 2020), and the abilities to learn without the support of a teacher are important for their academic success (Kingsbury, 2014), even more during digital learning (Warschauer, 2007). Digital learning requires a higher degree of motivation and persistence because the learner must take greater responsibility for his/her learning process compared to face-to-face learning with direct instructions from a teacher (Hill & Wouters, 2010).

### Technology acceptance and resistance

The TAM is often used to assess peoples’ acceptance regarding new technologies and is the most applied theory in e-learning acceptance studies (Park, 2009; Šumak et al., 2011). Davis (1989) found that the acceptance of technologies depends on the perceived ease of use (PEOU) and perceived usefulness (PU). PEOU describes the extent to which a person believes that using a particular application is effortless. PU is defined as the extent to which a person believes that using an application would improve their performance. Both variables affect the attitude toward using (ATT), which in turn, impacts the behavioral intention to use (BI) an application.

The TAM has been able to test the acceptance of digital learning in connection with a wide variety of factors in the literature. These factors include circumstances, emotions, social factors, but especially characteristics of the tested system or technology. For example, Bhattarai and Maharjan (2020) found that social influence, accessibility, computer self-efficacy, infrastructure, and enjoyment have a significant influence on both, PEOU and PU. Hanif et al. (2018) confirmed that subjective norm, perception of external control, system accessibility, enjoyment, and result demonstrability have a positive effect on PEOU and PU. Lee et al. (2005) could extend the TAM by integrating perceived enjoyment as an influencing variable on ATT and BI. However, they found PEOU was unrelated to ATT in their study. Meanwhile, Tarhini et al. (2013) confirmed the influence of social norms and quality of work life on digital learning acceptance, Mohammadi (2015) found system quality and information quality to be the primary factors driving users’ intention and satisfaction toward digital learning. Vladova et al. (2021) furthermore confirmed the positive influence of time and learning flexibility and social isolation on PU. However, they did not find a significant positive effect of PEOU on PU. Šumak et al. (2011) conducted a meta-analysis and investigated the effect of satisfaction, anxiety, and system quality, among other factors, finding that the effects between TAM constructs depend on the type of user and technology. Al-Azawei et al. (2017) integrated self-efficacy and learning styles into their TAM research.

The literature shows that various personal factors such as learning style, anxiety, or satisfaction have been used to describe the acceptance of digital learning. However, the impact of dispositions, like digital competences, self-organization, and independent learning abilities have not been investigated by other researchers before.

Another possibility to extend the TAM offers the resistance to digital learning. If the acceptance of digital learning is seen as the success of the implementation of digital learning technologies, the resistance to using these technologies can be described as the opposite of acceptance (Marakas & Hornik, 1996). Kim and Kankanhalli (2009) investigated possible reasons for the user resistance to information systems (IS) implementation and based their study on the status quo bias theory (Samuelson & Zeckhauser, 1988) and the equity implementation model (Joshi, 1991). They see user resistance as one of the main reasons why the implementation of a new IS fails and leads to people preferring the status quo (Kim & Kankanhalli, 2009). In the digital learning context, the status quo refers to face-to-face learning as opposed to digital learning, which represents a new alternative or change.

## Hypotheses development

**Fig. 1:**
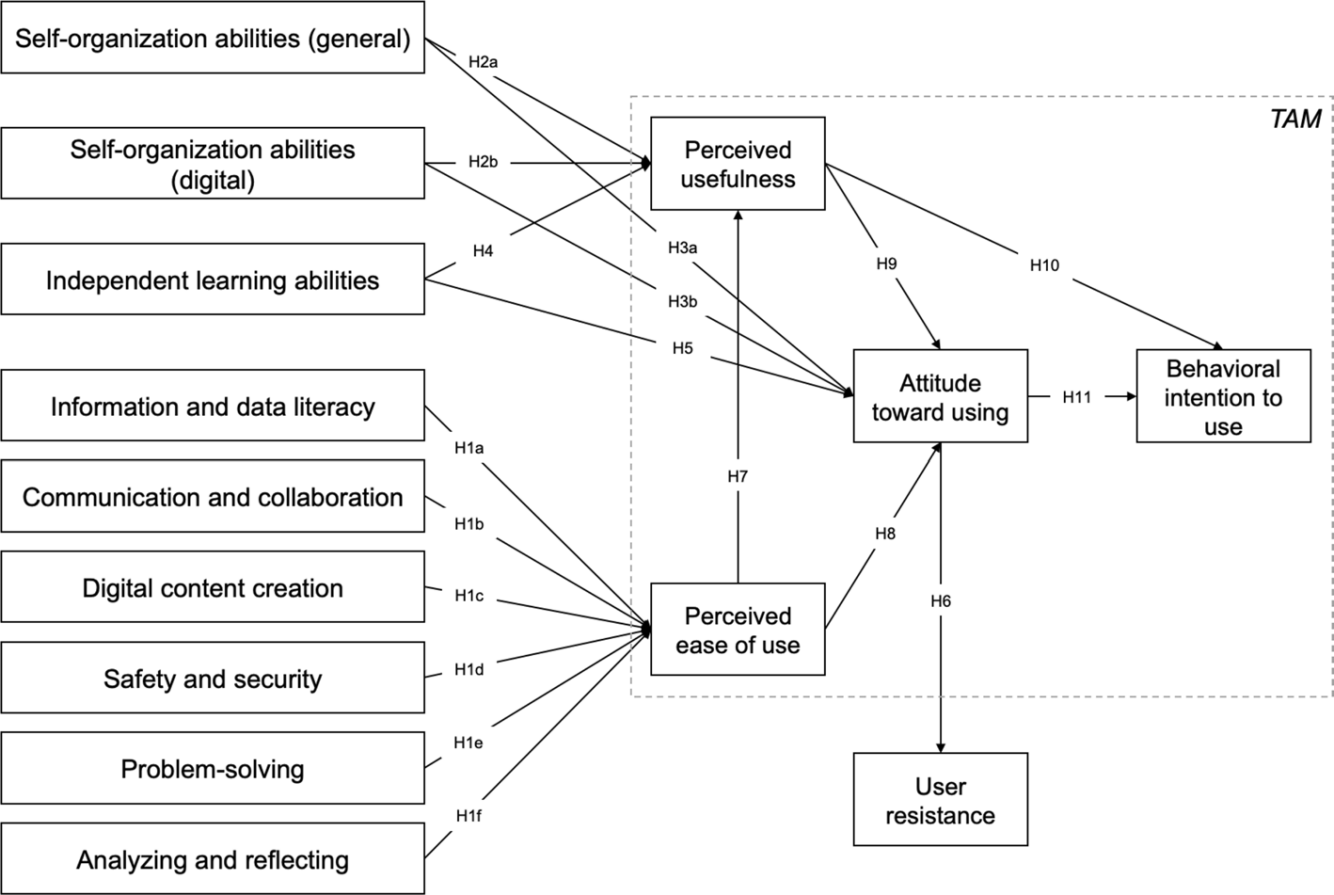
Research model

Building on this theoretical background of the constructs of digital competences, self-organization and independent learning abilities, user resistance, and constructs of the TAM, we can formulate a model of the influence of the different dispositions of students on their acceptance of digital learning and the effect of this acceptance on their resistance to digital learning. This influence will be derived in the following subsections. Figure 1 presents a summary of the hypotheses in the form of a research model.

### Digital competences

Although current students are generally considered to be technically savvy and experienced, they often lack digital competences, namely information and data literacy, communication and collaboration, digital content creation, safety and security, problem-solving, and analyzing and reflecting (Rubach & Lazarides, 2021). These competences are essential to successfully participate in digital learning activities (Anthonysamy et al., 2020; Littlejohn et al., 2012; Martzoukou et al., 2020; Muresan & Gogu, 2013; Tenku Shariman et al., 2014). Martzoukou et al. (2020) determine the perceived digital competences of students and a lack of different competences including information literacy (particularly issues regarding referencing information sources), digital creation (e.g., coding and programming skills), digital research, and identity management. Additionally, students have difficulties searching for information in an academic context (Coker, 2020; Littlejohn et al., 2012; Martzoukou et al., 2020; Shopova, 2014; Tenku Shariman et al., 2014). These problems mainly include a lack of evaluative and critical abilities (Littlejohn et al., 2012), that is, they usually only use one search engine for their research without advanced search strategies (Martzoukou et al., 2020), they lack the skills to make use of an electronic library or databases and the abilities to assess the quality of websites and interpret references (Coker, 2020; Shopova, 2014). Furthermore, they cannot estimate the validity and value of information and show difficulties when sharing information via digital technologies, for instance, regarding copyright issues (Tenku Shariman et al., 2014).

Digital competences go beyond operational skills and knowledge about how to use a certain technology or information (Coker, 2020), and include also the ability to use them critically and efficiently (Shopova, 2014). These difficulties in digital learning are compounded by a lack of support from teachers, leading to academic achievements being reduced by digital learning, for instance, if the learning achievements are classified as worse due to information of poor quality being used or a lack of critical assessment. If students do not know how to use the right information critically due to a lack of necessary digital competences, they would experience the use as difficult and would need to invest more time into learning how to use digital technologies properly to achieve satisfactory results. Similar problems can be applied to the other demanded digital competences in digital learning (see “Digital learning and students' dispositions” section). On the one hand, this necessary additional effort and cumbersome learning process could lead to digital technologies for learning being perceived as less easy to use. On the other hand, the presence of the different digital competences should mean that students do not have difficulty applying these competences.

- *H1a: Information and data literacy competences of students have a positive influence on their perceived ease of using digital technologies for learning.*
- *H1b: Communication and collaboration competences of students have a positive influence on their perceived ease of using digital technologies for learning.*
- *H1c: Digital content creation competences of students have a positive influence on their perceived ease of using digital technologies for learning.*
- *H1d: Safety and security competences of students have a positive influence on their perceived ease of using digital technologies for learning.*
- *H1e: Problem-solving competences of students have a positive influence on their perceived ease of using digital technologies for learning.*
- *H1f: Analyzing and reflecting competences of students have a positive influence on their perceived ease of using digital technologies for learning.*

### Self-organization abilities

When digital learning can take place almost exclusively from the comfort of one’s own home, self-organization abilities are strongly demanded since students are often interrupted or exposed to many distractions, such as parcel deliveries, roommates, or simply not being able to focus properly, that interfere with efficient learning and correct understanding of the learning content (Owusu-Fordjour et al., 2020). Additionally, students have difficulties in structuring their routines due to the lack of scheduled classes (Costa et al., 2018; Klein et al., 2021) and keeping the learning pace they are used to from face-to-face learning (Klein et al., 2021). Digital learning demands students to have a high level of motivation, time management, and focus (Hameed et al., 2008). Overall, students seem to have serious difficulties, especially with time management (Muresan & Gogu, 2013; Uzun et al., 2013) and self-organization abilities in general during digital learning (Muresan & Gogu, 2013). Furthermore, they seem to be aware of their self-organization problems as they prefer digital technologies for learning that improve their self-organization abilities, for example, with the help of visual progress bars (Noskova et al., 2021), which shows that they demand additional help for self-organization when learning digitally.

Reinforcing the extent of difficulties with self-organization during digital learning, Bernard et al. (2004) and Klein et al. (2021) were able to demonstrate that a higher level of self-organization has a positive effect on learning performance. However, if students cannot reach the expected performance due to the aforementioned obstacles, it can lead to them to question the usefulness of digital learning. On the contrary, if higher degrees of self-organization abilities can lead to improved performance in digital learning, this could increase PU. For our hypotheses, we differentiate between general self-organization abilities and self-organization abilities during digital learning (cf. Klein et al., 2021) to further distinguish the impact of the specific challenges during digital learning.

- *H2a: General self-organization abilities have a positive influence on the perceived usefulness of digital learning.*
- *H2b: Self-organization abilities during digital learning have a positive influence on the perceived usefulness of digital learning.*

In addition to the perceived usefulness, high self-organization abilities are associated with a positive attitude of students toward digital learning in general (Klein et al., 2021; Uzun et al., 2013). Possible reasons could include that self-organization in the digital learning process is also challenged regarding the reconciliation of private life and studies. Due to the need to perform most of the study-related activities in private circumstances, those boundaries are difficult to maintain (Coker, 2020). This limitation means that students who do not manage to plan their time accordingly experience a higher restriction of their free time due to digital learning. Furthermore, a greater ability to self-organize facilitates better planning for how to reach a certain goal (Kostromina, 2013). This would indicate that students with high overall objectives of their academic career, for instance, to get a certain job, but low self-organization abilities, face the risk of not reaching those goals due to having to learn digitally.

Both the restriction of private time as well as the possible denial of certain objectives go beyond difficulties during the explicit learning process and could lead to students with higher self-organization abilities having a more positive overall attitude toward digital learning.

- *H3a: General self-organization abilities have a positive influence on the attitude toward digital learning.*
- *H3b: Self-organization abilities during digital learning have a positive influence on the attitude toward digital learning.*

### Independent learning abilities

Hockings et al. (2018) and Deepwell and Malik (2008) investigated the abilities and thoughts of students toward independent learning, finding certain difficulties. The authors found that during independent learning, some students still rely on teachers for guidance in the learning process, for instance, by demanding faster feedback from the teacher or more instruction. In addition, some students solely relied on the reading supplied but acknowledged that advanced reading through the library portals was needed for better marks (Deepwell & Malik, 2008). Furthermore, Hockings et al. (2018) reported that students who were unsure what they needed to do only completed the assessment tasks they were given and perceived independent learning more like homework, thus not making use of the full potential of independent learning. Another challenge regarding independent digital learning is the missing engagement of teachers in the learning process, which is vital, particularly for current students (Mosca et al., 2019). This challenge is again linked to the lack of extrinsic motivation from teachers or students who could motivate each other in a live setting.

In general, aspects of independent learning are challenging for students even though they play a vital role in the success of digital learning. Lower results could lead to digital learning being perceived as less useful than traditional learning, which involves more supervision by an instructor.

- *H4: Independent learning abilities have a positive influence on the perceived usefulness of digital learning.*

Although digital learning offers a higher degree of freedom for the learner, especially in online spaces, Costa et al. (2018) and Hockings et al. (2018) reported that while some students are embracing the freedom during independent learning, some felt overwhelmed and anxious and were unsure about what was expected of them. A potential cause could be an overall lack of guidance on how to learn independently and use technologies during independent learning (Deepwell & Malik, 2008). This is a significant limitation, as a lack of mentorship and guidance by teachers while digital learning can lead to students being abandoned and left alone in the learning process (Warschauer, 2007). Moreover, some students lack the experience of independent learning and therefore prefer to learn in traditional ways (Noskova et al., 2021).

On the one hand, an overwhelming and anxious feeling of acting autonomously in the learning process without knowing what is expected and precisely what to do could lead to a lower attitude toward digital learning in general. On the other hand, students with higher independent learning abilities who can take advantage of the opportunities for freedom offered by digital learning could have a better attitude toward digital learning.

- *H5: Independent learning abilities have a positive influence on the attitude toward digital learning.*

### Resistance to digital learning

Students’ demonstrated preference for face-to-face over digital learning (Radha et al., 2020) offers the possibility to apply the status quo bias theory and further investigate the possible resistance to digital learning. In their theory, Samuelson and Zeckhauser (1988) distinguish three possible reasons for the status quo bias: rational decision-making based on transition costs or uncertainty, cognitive misperceptions, and psychological commitment. In particular, the first reason can be applied to digital learning and the related circumstances of students who have already been mentioned above. In this context, the transition costs could represent the additional efforts students are burdened with by switching from traditional to digital learning. These costs include changing familiar routines and learning how to use modern technologies and incorporate them into their learning process. Furthermore, Hockings et al. (2018) discovered that students tend to feel overwhelmed and anxious when studying individually and are unsure of their teachers’ expectations. Learning digitally also means solving problems independently and accepting an increase in responsibility for their own learning performance. This burden can be connected to uncertainty, which influences the status quo bias (Samuelson & Zeckhauser, 1988).

These factors can also be adopted to the equity implementation model. Joshi (1991) describes that the resistance to change during the implementation of new technology systems is based on equity theory, which states that people resist change due to their assessment of perceived inputs and outputs and the fairness of this exchange. Within the model, several factors are mentioned that can lead to an increase or decrease of inputs and outcomes. Factors that influence the increase in inputs and the decrease in outcomes include, for example, the effort in learning a new system, the need to spend more time, fear of the unknown (e.g., failure) and the resulting anxiety, as well as the potential failure to learn and adopt the new system (Joshi, 1991).

Applying these factors to digital learning and the use of corresponding technologies supports the perceived inequity of changing from face-to-face to digital learning. In addition, the status quo bias indicates a negative attitude toward the use of digital technologies for learning. According to the theories discussed, a poor attitude leads to user resistance. In contrast, a positive attitude would not lead to preferring the status quo and wanting to reject digital learning.

- *H6: The attitude toward digital learning has a negative influence on the resistance to digital learning.*

### Technology acceptance model

According to Davis (1989), PEOU is positively related to PU and ATT. In addition, PU has a positive influence on ATT and BI, and ATT is positively related to BI. To validate the TAM in the context of this study and consistent with prior research (cf. Lee et al., 2005; Vladova et al., 2021), the following hypotheses are tested:

- *H7: The perceived ease of using digital technologies for learning has a positive influence on the perceived usefulness of digital learning.*
- *H8: The perceived ease of using digital technologies for learning has a positive influence on the attitude toward digital learning.*
- *H9: The perceived usefulness of digital learning has a positive influence on the attitude toward digital learning.*
- *H10: The perceived usefulness of digital learning has a positive influence on the behavioral intention to use digital technologies for learning.*
- *H11: The attitude toward digital learning has a positive influence on the behavioral intention to use digital technologies for learning.*

## Materials and methods

### Study design

To test the research model, an online survey during the period from September to October 2021 was conducted using the website Prolific. We used Prolific as the website to conduct the survey because the platform is specifically designed for scientific studies, accurately informs only registered participants about the purpose of the studies, and professionally handles the implementation with certain restrictions and rules (Palan & Schitter, 2018). In addition, the platform allows to generate a global and representative sample in order to draw generally valid conclusions and not to limit the validity of the answers to individual regions. Participants were required to have student status on either an undergraduate (BA/B.Sc/similar) or a graduate (MA/M.Sc/similar) level, which excludes, for example, doctoral students, as they do not attend the usual lectures and seminars and thus do not experience digital learning to the same extent. Other than that, the study did not require any further limitations on the sample. All participants offered informed consent for the use of their responses. All questions were designed as mandatory, which prevented missing values. Furthermore, the survey included two attention-check questions.

### Measures and procedure

Prolific provided the demographic data of the participants. In the survey, we asked general questions about studying and the experience with digital learning (cf. the full set of questions in Appendix A). To evaluate the constructs of the research model, slightly modified pretested scales were applied, evaluated with a five-point Likert scale ranging from 1 (strongly disagree) to 5 (strongly agree).

Concerning the concepts of the TAM, the scales for PU, PEOU, and BI were applied from Lee et al. (2005). As their scale to evaluate ATT cannot be applied to a five-point Likert scale and the wording of the individual items is quite general, the items by Vladova et al. (2021), who also based the development of their scales on Lee et al. (2005), were applied to this study. Additionally, one item of the scale for ATT was substituted by a self-developed item to match the particular context of the study, since the item used by Vladova et al. (2021) specifically targeted the context of the COVID-19 pandemic. For sources of the remaining scales, see Table 1. Furthermore, most of the scales were slightly modified to match the wording and overall context of this study.

**Table 1:** Measurement scales and sources

Concept/Context	Construct	Measurement scale source
TAM	Perceived usefulness	Modified from Lee et al. (2005)
	Perceived ease of use	Modified from Lee et al. (2005)
	Attitude toward using	Modified and further developed from Vladova et al. (2021)
	Behavioral intention to use	Modified from Lee et al. (2005)
Digital competences	Information and data literacy	Adopted from Rubach and Lazarides (2021)
	Communication and collaboration	Adopted from Rubach and Lazarides (2021)
	Digital content creation	Adopted from Rubach and Lazarides (2021)
	Safety and security	Adopted from Rubach and Lazarides (2021)
	Problem-solving	Adopted from Rubach and Lazarides (2021)
	Analyzing and reflecting	Adopted and modified from Rubach and Lazarides (2021)
Self-organization abilities	General self-organization abilities	Adopted from Klein et al. (2021)
	Self-organization abilities during digital learning	Adopted and modified from Klein et al. (2021)
Independent learning abilities	Independent learning abilities	Adopted from Macaskill and Taylor (2010)
User resistance	User resistance	Modified from Kim and Kankanhalli (2009)

A pretest was conducted in August 2021 with 45 students to test the general comprehensibility. After the evaluation of the pretest and the respective feedback, minor changes were conducted: We rephrased additional information in two of the questions and substituted one of the items in the scale for ATT.

The descriptive statistics of the participants are illustrated in Table 2, showing a diverse sample regarding sex, nationality, and study subject.

**Table 2:** Descriptive statistics of participants

Demographic variable	Data
Sex	
Female	196 (56%)
Male	154 (44%)
Age (years) (mean = 23.62, SD = 5.955)	
< 20	51 (14.6%)
20–24	210 (60%)
25–30	63 (18%)
> 30	26 (7.4%)
Nationality	
South Africa	113 (32.3%)
Portugal	36 (10.3%)
Italy	30 (8.6%)
Poland	23 (6.6%)
Spain	18 (5.1%)
Other	130 (37.1%)
Current education level	
Undergraduate degree	230 (65.7%)
Graduate degree	120 (34.3%)
Study subject	
Engineering	31 (8.9%)
Computer Science	29 (8.3%)
Accounting	22 (6.3%)
Computing (IT)	20 (5.7%)
Business	16 (4.6%)
Other	232 (66.3%)

### Data analysis

The data was prepared and analyzed using IBM SPSS Statistics, version 27. 10 of the data sets were deleted, because the participants failed an attention check that asked them to select a certain response (e.g. “To make sure you are paying attention, please click “never” here.”). Regression analysis using the OLS method was applied to test the individual models. The mean values of the respective items were used to calculate the model constructs.

#### Measurement validity

To measure the validity of all constructs, we conducted two separate approaches. Since most of the pretested scales were slightly modified and adjusted to the context of this study, we conducted an exploratory factor analysis (EFA). We first used the Kaiser–Meyer–Olkin (KMO) criterion to test whether the correlation matrices were suitable for factor analysis. All of them reached a value of 0.5 and could therefore be used for the EFA (cf. Ferguson & Cox, 1993). We then employed an EFA for the items of the TAM (KMO = 0.842), items explaining the different constructs of digital competences (KMO = 0.876), and a final EFA for the remaining constructs (KMO = 0.862) (cf. Appendix B) to ensure their suitability to measure connected items through the items’ factor loadings. To achieve a reduction and replication of the data structure using as few uncorrelated factors as possible, and thus creating a pattern of similarity for each variable, we used a principal component analysis (cf. Abdi & Williams, 2010) with a varimax rotation on the items. Factors with strong loadings (≥ 0.5, cf. Costello & Osborne, 2005) on the constructs were considered for further analysis. This led to 10 dropped items as they did not match the respective scales to a significant extent. Details about the conducted EFA and the handling of deleted items due to low factor loadings are presented in Appendix B. All other factor loadings ranged from 0.516 to 0.867. Apart from that, the results show that the construct problem-solving should be addressed using two different factors. However, the division of this construct matches the categorization of the measurement scale source Rubach and Lazarides (2021), who also divided this competence area after the EFA into the two subscales *operation and usage* (problem-solving I), and *comprehension and development* (problem-solving II). Accordingly, we have adjusted the list of hypotheses, which can be viewed in Appendix C. Afterward, the internal validity of the scales was measured using the reliability coefficient Cronbach’s alpha (CA) (see Appendix B). Only the scale to measure independent learning abilities missed the fulfilment of internal validity (≥ 0.7, cf. Tavakol & Dennick, 2011) by a narrow margin.

#### Descriptive information on model constructs

Table 3 presents the descriptive information for the model constructs, i.e., the different dependent and independent variables. Concerning the TAM core, PEOU was rated the highest (avg. 3.90, SD = 0.731), while ATT received the lowest results (avg. 2.66, SD = 1.003). Examining the digital competences, the participants rated their problem-solving competences related to operation and usage with the highest scores (avg. 4.21, SD = 0.511) and their problem-solving competences related to comprehension and development with the lowest scores (avg. 3.37, SD = 0.887). Overall, and compared to the other constructs, the participants assessed their digital competences the highest. The general self-organization abilities were evaluated higher (avg. 3.65, SD = 0.833) than the self-organization abilities during digital learning (avg. 2.92, SD = 0.994), which appear relatively low, also in comparison to the assessed independent learning abilities (avg. 4.17, SD = 0.483). The resistance to digital learning received a medium assessment on the five-point Likert scale (avg. 2.55, SD = 0.952).

**Table 3:** Descriptive information on model constructs

Construct	Min	Max	Mean	SD
Perceived usefulness	1	5	3.41	0.929
Perceived ease of use	1	5	3.90	0.731
Attitude toward using	1	5	2.66	1.003
Behavioral intention to use	1	5	3.86	0.777
Information and data literacy	3	5	4.17	0.502
Communication and collaboration	2	5	4.12	0.713
Digital content creation	1	5	3.88	0.734
Safety and security	2	5	4.05	0.617
Problem-solving I	2	5	4.21	0.511
Problem-solving II	1	5	3.37	0.887
Analyzing and reflecting	1	5	3.80	0.616
General self-organization abilities	1	5	3.65	0.833
Self-organization abilities during digital learning	1	5	2.92	0.994
Independent learning abilities	3	5	4.17	0.483
User resistance	1	5	2.55	0.952

## Results

**Fig. 2:**
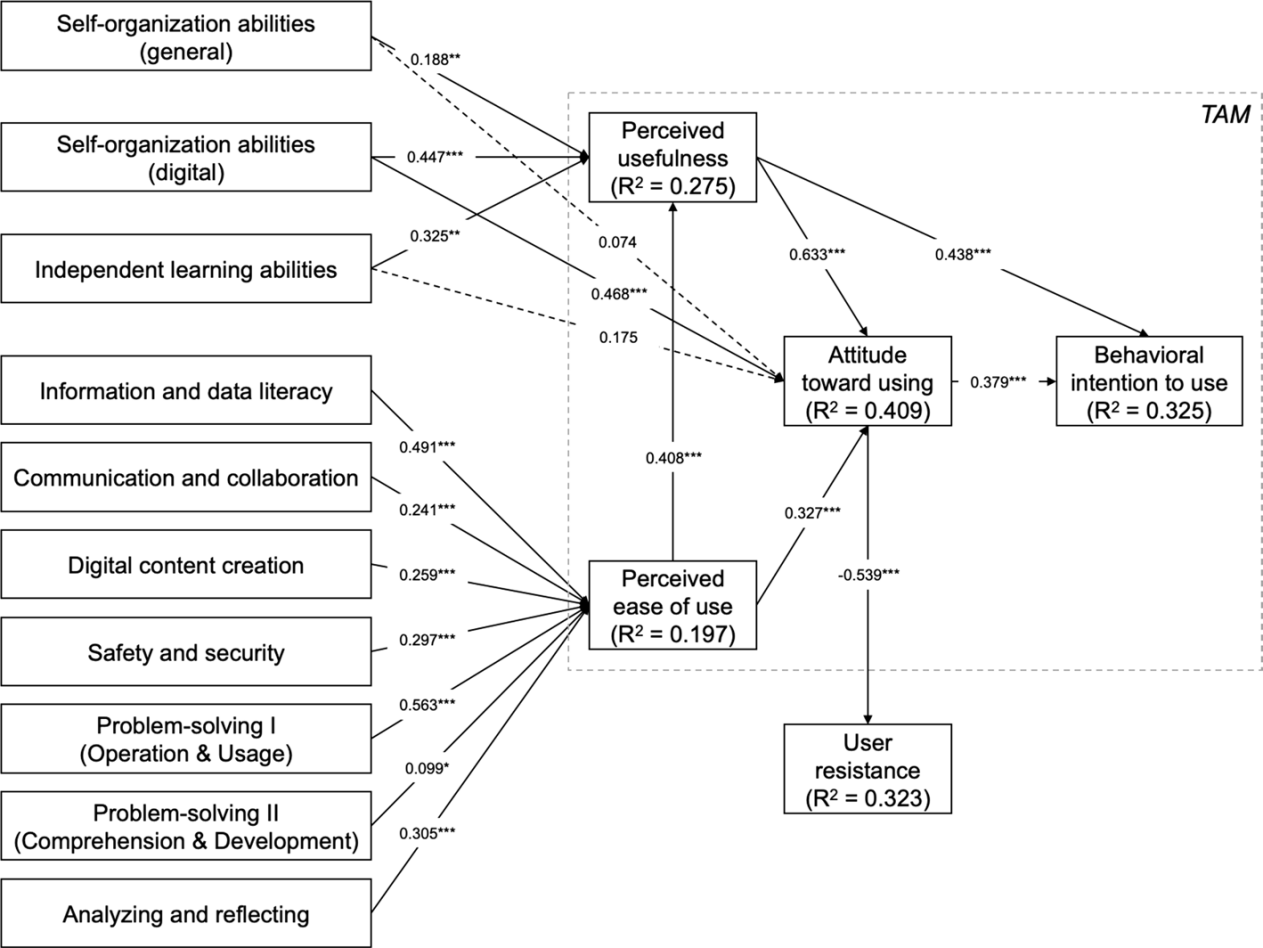
Hypothesis test results. * Significant at the 0.05-level, ** significant at the 0.01-level and *** significant at the 0.001-level.  represents a significant link,  represents an insignificant link

Figure 2 shows all the results of the regression analysis with its coefficients, describing the relationship between the independent and dependent variables, and the significance of this relationship in order to test the hypotheses. That is, the closer the coefficient is to one, the stronger the relationship between the two variables. Table 4 offers further details on the coefficients for the variables and the constants of the individual models. For mathematical details on regression analysis, see Seber and Lee (2012). For all constructs, the survey data was used with the adjusted scales after the EFA. The results of the hypothesis tests show that all hypotheses, with the exceptions of H3a (General self-organization abilities ➔ ATT) and H5 (Independent learning abilities ➔ ATT), could be verified.

**Table 4:** Summary of hypothesis tests

Hypothesis	Coefficient constant	Coefficient independent variable	P-value	Test result
H1a: Information and data literacy ➔ PEOU	1.847	0.491	0.000	Confirmed
H1b: Communication and collaboration ➔ PEOU	2.593	0.241	0.000	Confirmed
H1c: Digital content creation ➔ PEOU	2.894	0.259	0.000	Confirmed
H1d: Safety and security ➔ PEOU	2.694	0.297	0.000	Confirmed
H1e: Problem-solving I ➔ PEOU	1.528	0.563	0.000	Confirmed
H1f: Problem-solving II ➔ PEOU	3.564	0.099	0.025	Confirmed
H1g: Analyzing and reflecting ➔ PEOU	2.741	0.305	0.000	Confirmed
H2a: General self-organization abilities ➔ PU	2.723	0.188	0.002	Confirmed
H2b: Self-organization abilities during digital learning ➔ PU	2.103	0.447	0.000	Confirmed
H3a: General self-organization abilities ➔ ATT	2.395	0.074	0.252	Not confirmed
H3b: Self-organization abilities during digital learning ➔ ATT	1.300	0.468	0.000	Confirmed
H4: Independent learning abilities ➔ PU	2.053	0.325	0.002	Confirmed
H5: Independent learning abilities ➔ ATT	1.937	0.175	0.116	Not confirmed
H6: ATT ➔ User resistance	3.990	− 0.539	0.000	Confirmed
H7: PEOU ➔ PU	1.817	0.408	0.000	Confirmed
H8: PEOU ➔ ATT	1.389	0.327	0.000	Confirmed
H9: PU ➔ ATT	0.507	0.633	0.000	Confirmed
H10: PU ➔ BI	2.372	0.438	0.000	Confirmed
H11: ATT ➔ BI	2.856	0.379	0.000	Confirmed

With the first hypotheses, we tested the effect of different digital competences on PEOU. Using linear regressions of each competence area, we found that all these competence areas had a significant positive effect on PEOU. Observing the coefficients in Table 4, all the effects are highly significant apart from the effect of problem-solving II (comprehension and development) on the PEOU, which is significant at the 0.05-level. The effects of all digital competences explained 19.7% of the variance in PEOU in the model test (see Table 5).

**Table 5:** Multiple linear regression explaining perceived ease of use

Construct	R^2^	Coefficient
	0.197	
Information and data literacy		0.236**
Communication and collaboration		0.046
Digital content creation		0.095
Safety and security		0.097
Problem-solving I (Operation and usage)		0.322***
Problem-solving II (Comprehension and development)		− 0.075
Analyzing and reflecting		0.040

^*^Significant at the 0.05-level, ** significant at the 0.01-level and *** significant at the 0.001-level

Furthermore, the effects of self-organization abilities in general, self-organization abilities during digital learning, and independent learning abilities on PU and ATT were measured. All three constructs show a significant positive effect on PU (Table 4). However, only the self-organization abilities during digital learning have a significant positive effect on ATT. Although the general self-organization and independent learning abilities show a small positive effect, it is not significant. In addition to the hypotheses regarding the tested influencing factors on PEOU and PU, all hypotheses connecting the constructs inside the TAM could be confirmed. The effects of PEOU, independent learning abilities, general self-organization abilities, and self-organization abilities during digital learning explained 27.5% of the variance in PU in the model test (see Table 6). The effects of PEOU, PU, independent learning abilities, general self-organization abilities, and self-organization abilities during digital learning explained 40.9% of the variance in ATT (see Table 7). Moreover, the effects of ATT and PU explained 32.5% of the variance in BI (see Table 8).

**Table 6:** Multiple linear regression explaining perceived usefulness

Construct	R^2^	Coefficient
	0.275	
Perceived ease of use		0.265***
Independent learning abilities		0.061
General self-organization abilities		− 0.065
Self-organization abilities during digital learning		0.415***

^*^Significant at the 0.05-level, ** significant at the 0.01-level and *** significant at the 0.001-level

**Table 7:** Multiple linear regression explaining attitude toward using

Construct	R^2^	Coefficient
	0.409	
Perceived ease of use		0.038
Perceived usefulness		0.494***
Independent learning abilities		− 0.059
General self-organization abilities		− 0.179**
Self-organization abilities during digital learning		0.316***

^*^Significant at the 0.05-level, ** significant at the 0.01-level and *** significant at the 0.001-level

**Table 8:** Multiple linear regression explaining behavioral intention to use

Construct	R^2^	Coefficient
	0.325	
Attitude toward using		0.214***
Perceived usefulness		0.303***

^*^ Significant at the 0.05-level, ** significant at the 0.01-level and *** significant at the 0.001-level

In addition to those influencing factors, we analyzed the effect of the students’ ATT on the potential resistance to digital learning. The hypothesized negative effect could be confirmed at a highly significant level (Table 4). The effect of ATT explained 32.3% of the variance in user resistance in the model test.

## Discussion and implications

### Discussion of the results

The aim of this study was to investigate the influence of students’ digital competences, self-organization, and independent learning abilities on their acceptance of digital learning. The results allow us to gain deeper knowledge about the rationale behind the aversion to digital learning. We validated the hypotheses in the context of this study and thus answered the research question: Students’ digital competences, self-organization, and independent learning abilities influence their acceptance of digital learning positively. Using the TAM and its different constructs, these relationships could be deeper scrutinized. Accordingly, digital competences influence the acceptance through the perceived ease of using digital technologies for learning, while self-organization and independent learning abilities influence the acceptance through the perceived usefulness of digital learning. Details are presented in the following subsections.

#### The acceptance of digital learning

All hypotheses connecting the TAM constructs could be confirmed, most importantly the significant positive effects of PU and PEOU on ATT, which confirms results of existing studies using the TAM in the digital learning context (e.g., Liu et al., 2005; Šumak et al., 2011). However, Lee et al. (2005), who were the main source of the TAM scales, could not confirm a positive influence of PEOU on ATT. This finding might be connected to the adjustments we made to the scale measuring ATT based on Vladova et al. (2021), who also confirmed a positive connection between PEOU and ATT.

#### The influence of digital competences on acceptance of digital learning

The results show that students’ digital competences, that is, competences concerning information and data literacy, communication and collaboration, digital content creation, safety and security, problem-solving, and analyzing and reflecting, all have a positive effect on the perceived ease of using digital technologies for learning. This effect in turn influences the attitude toward digital learning positively, which indicates that students with higher digital competences endorse the growing change from face-to-face to digital learning. This result implies that students with higher overall digital competences have a greater acceptance of digital learning.

The individual effects of different digital competence areas on PEOU were investigated in more detail. All competence areas show a significant positive effect on PEOU. The lowest effect showed problem-solving competences regarding comprehension and development. Examining the items, it becomes clear that those competences address special skills regarding algorithmic structures and the functioning of digital systems (see Table 16). Regarding content, this competence area thus shows no direct connection to digital competences that are essential during digital learning, which explains why the connection between these two constructs is weaker than others.

Although digital competences are essential for participation in digital learning (Almerich et al., 2020), several studies show that students often lack these necessary skills (e.g., Martzoukou et al., 2020). Possible reasons for the positive relationship between digital competences and PEOU include that students lacking digital competences have more difficulties using the technologies in digital learning in the required way. This leads to students having to invest more effort and time into digital learning to keep the same level of performance compared to face-to-face learning. For example, insufficient digital competences due to the lack of criticality about sources in digital environments might lead to worse grades. This result in turn means students must invest more time into searching for trustworthy information. This scenario can lead to students preferring face-to-face learning even more, for example, by searching for information in a physical library at the university campus with fewer demands for digital competence.

#### The influence of self-organization abilities on acceptance of digital learning

A positive influence of self-organization abilities on the acceptance of digital learning through PU could be demonstrated in this work. Thus, the results support the literature regarding the positive effect of self-organization abilities such as time management and self-discipline on academic success (Claro & Loeb, 2019; Klein et al., 2021; Kostromina, 2013), particularly during digital learning (Anthonysamy et al., 2020). More specifically, students with higher self-organization abilities, both overall and during digital learning, perceive digital learning to be more useful. This effect on the construct PU implies that students think that digital learning has a positive effect on their performance.

However, only self-organization abilities in particular during digital learning positively influence ATT. This indicates that when students are confronted with a direct comparison between digital and face-to-face learning (construct ATT), they do not necessarily prefer digital learning despite having higher overall self-organization abilities. The descriptive results of the constructs (Table 3) represent one explanatory approach: The participants rated ATT lower than the other TAM constructs, which explains the differing impact on PU and ATT. Furthermore, they rated their self-organization abilities during digital learning lower than the general self-organization abilities. This finding is an indicator for self-organization abilities being even further challenged during digital learning. In turn, the results suggest that lower self-organization abilities during digital learning enhance their aversion to digital in comparison to face-to-face learning. Accordingly, these abilities represent a crucial factor that must be considered for the success of digital learning. Suggestions for practical implications are presented in “Practical implications” section.

#### The influence of independent learning abilities on acceptance of digital learning

We were able to confirm a positive effect of independent learning abilities on PU and, therefore, on the acceptance of digital learning. Our findings are in line with prior literature on the positive effect of independent learning abilities on academic success (Kingsbury, 2014; Warschauer, 2007). Nevertheless, students with higher independent learning abilities do not necessarily prefer digital over face-to-face learning, as represented by ATT. Possible explanations are that direct mentorship from and contact with instructors and other students is still considered important, despite taking advantage of digital learning and being able to learn without direct instructions. This learning process includes students motivating each other and generating the feeling of performing as a group rather than as an individual who is left on his or her own. The implication is that independent learning abilities go beyond simply being able to reach certain academic goals but also include social relationships and interpersonal aspects and needs that are better fulfilled through face-to-face learning.

#### The influence of attitude toward digital learning on resistance to digital learning

To further investigate the consequences of a negative attitude toward digital learning, we measured the impact on resistance to digital learning, which is mainly based on the status quo bias theory (Samuelson & Zeckhauser, 1988). We could confirm that a negative ATT could even lead to students resisting participation in digital learning activities. While ATT measures a tradeoff between digital and face-to-face learning, data shows that students tend to reject the replacement of traditional with digital learning and rather prefer the status quo. The main reasons for this resistance could include feared additional efforts during digital learning, represented by the PEOU and its positive relationship to ATT (see Fig. 2), and the uncertainty and doubts of students toward digital learning (Akcil & Bastas, 2020). Examining the research model, digital competences, self-organization, and independent learning abilities seem to be indicators for those uncertainties.

### Theoretical implications

Our findings show the positive influence of digital competences, self-organization, and independent learning abilities on the acceptance of digital learning, and contribute to the body of literature on digital learning acceptance. By doing so, we fill important research gaps: The results show deeper insights into how students’ dispositions impact the acceptance of digital learning, and therefore, the success of digital learning in general (Kreidl, 2011).

We extended the TAM in the context of digital learning as the core of our research model. The different constructs of the TAM, mainly PEOU and PU, allowed us to further investigate the connections behind the influence of the dispositions on student acceptance. Additionally, we contributed to the literature on TAM by applying personal dispositions as influencing factors, especially since TAM research in the digital learning context is mainly characterized by functions and components of technologies and not personality traits (compare “Technology acceptance and resistance” section). Furthermore, we adjusted the original scale to measure ATT in Lee et al. (2005) and Vladova et al. (2021) to match the more general context of digital learning in higher education and were able to validate the new scale. As we were able to confirm all hypotheses connecting the TAM constructs, we can suggest using this new scale when measuring digital learning acceptance with TAM.

As Kim and Kankanhalli (2009) based their study on the status quo bias theory (Samuelson & Zeckhauser, 1988) and the equity implementation model (Joshi, 1991), we observed similarities in the derivation of these theories and possible reasons for the aversion to digital and the preference for face-to-face learning (see “Resistance to digital learning” section). By using these theories, we found first evidence that they can be applied in the digital learning context, particularly in the change from face-to-face (status quo) to digital learning.

### Practical implications

The results of this study form a basis for recommendations for action for lecturers and decision-makers of higher education institutions to strengthen students’ acceptance of digital learning. Since digital competences, self-organization, and independent learning abilities have a significant impact on students’ acceptance, they should form an essential part of strategies for digital learning success, such as for the conceptual preparation of lectures or seminars and planning the use of certain technologies.

Related research on digital competences indicates that many students have problems with digital competences in the higher education context (cf. Littlejohn et al., 2012; Martzoukou et al., 2020; Tenku Shariman et al., 2014). However, most universities have yet failed to map digital competences of their students and implement approaches to study programs or curricula to facilitate them. The few activities that aim to develop digital competences do not address the individual needs of the students but rather see them as a homogenous group. Additionally, universities that are already implementing activities to support students in developing digital competences often aim these initiatives at basic competences (Martzoukou et al., 2020), whereas advanced digital competences are needed to successfully participate in digital learning activities and be a competitive candidate on the labor market (European Commission, 2021). Furthermore, within the generation of digital natives there are still students disinterested in the use of digital technologies (Martzoukou et al., 2020) or frequently fail to use them in other contexts than merely leisure time (Littlejohn et al., 2012). Thus, universities should focus on enhancing the digital competences of their students, clearly communicate the requirements for academic standards, and offer individual support.

Instead of leaving the students completely on their own, lecturers should enhance the self-organization abilities of students and support them in more organized studying. For example, keeping a schedule for online lectures and seminars with regular assignments and deadlines. In addition, students should be animated to help and motivate each other, for example, through group work or discussions. Furthermore, instructors should be careful not to increase the amount of learning content and activities during digital learning because they assume that students have more available time. Especially with digital learning, it must be ensured that students have enough time for leisure and can clearly separate that from study activities. Regarding independent learning abilities, students need to be given the impression that they do not study completely on their own but can receive support and instructions when needed, for instance, by enhanced group work or scheduled feedback meetings with instructors. It can help to use as many interactive activities, methods, and tools as possible to keep in direct contact with the students.

### Limitations

This study has some limitations. First, to make statements about the population of all university students, the sample size is relatively small but sufficient to obtain robust and significant results. Furthermore, the sample is diverse regarding nationalities, genders, and fields of study. During the analysis, however, we did not consider this diversity further, as this was not in focus. Moreover, assessments of one’s own abilities and competences can be influenced by self-enhancement bias (Gosling et al., 1998). This means that when interpreting the study results, it is essential to consider that participants might have rated their competences and abilities higher than they actually are. Note that for data analysis, some of the scales, particularly scales for certain digital competences, had to be adopted after the EFA, which means that their informative value must be interpreted carefully. In our study, we clearly separated digital from face-to-face learning and therefore did not consider hybrid forms of these two learning formats.

## Conclusion and future research

Inspired by the restrained attitude of students toward the change from face-to-face to digital learning in higher education, we conducted a study to investigate the influence of students’ personal dispositions on their acceptance of digital learning. The results indicate that certain dispositions are crucial when developing strategies for digital learning. In particular, we were able to show that digital competences, self-organization, and independent learning abilities have a significant positive impact on students’ acceptance of digital learning. Furthermore, our results indicate that if students’ acceptance is absent, students could show resistance toward digital learning, which directly influences its success. We analyzed this impact using OLS regression analysis on an extensive research model with the technology acceptance model as the core to measure the acceptance of digital learning. This analysis contributed to further investigating the underlying factors that can lead to more positive student perceptions of digital learning. More importantly, three concrete reference values could and should be directly addressed in teaching practice to support the change from face-to-face to digital learning in higher education. Some recommendations have been formulated in the previous sections.

Based on those findings, future research can focus on explorative qualitative studies to investigate additional factors influencing the acceptance of digital learning, thereby addressing some of the limitations of this study. For example, we will consider different hybrid learning formats, using that approach to investigate precisely where students see advantages and disadvantages in digital learning in connection to which academic activities and digital technologies. These approaches will support gaining a deeper knowledge about what entails an ideal learning experience for students in a technology-supported setup. Moreover, we consider differences in demographics to extend our knowledge, for example, regarding the influence of digital competences of students on their acceptance of digital learning for various study subjects or different genders.

## Data Availability

The datasets used and/or analyzed during the current study are available from the corresponding author on reasonable request.

## Appendices

### Appendix A: Survey questions

See Tables

**Table 9:** Survey questions: General question about studies

In which semester are you studying in your current degree programme?
In which country is your university located?
Which study type do you classify yourself as?
Under normal circumstances (outside of the current COVID-19 pandemic), do the events within your studies (lectures, seminars etc.) mainly take place in presence at the university or online?
During the COVID-19 pandemic, do the events within your studies (lectures, seminars etc.) mainly take place in presence at the university or online?
How much is your total study-related weekly working time on average (incl. lecture duration with preparation and follow-up, seminars, group work etc.)?
Has this weekly working time changed due to the COVID-19 pandemic?
Please rate how often you use the following categories of technologies during your studies, e.g., during lectures/seminars, for group work, or for your personal study-related work, like the preparation of lectures or exams
Technologies to access and study learning materials (e.g., learning management systems such as Moodle, Canvas or Blackboard)
Technologies that enable learning collaboration (e.g., services that allow the simultaneous revision of a shared document or presentation like Google Docs or Office Online)
Technologies that enable learning communication (e.g., tools like Zoom, Skype or Slack)
Technologies for assessing learners and learning outcomes (e.g., online tests or websites like Kahoot and Mentimeter)
Technologies enabling a learning-by-doing approach through construction and programming (e.g., assembling and programming robotics)
Technologies for developing digital and multimedia literacy (e.g., multimedia tools such as video editing or image processing)
To make sure you are paying attention, please click “never” here

9,

**Table 10:** Survey questions: TAM

*Technology acceptance* The following questions relate to your personal experience of digital learning and the use of the associated technologies during your studies. As already mentioned, these technologies include e.g. technologies to access learning materials, to collaborate and communicate, to assess learning outcomes, and to develop digital literacy Please rate the following statements as honestly and sincerely as possible
Digital learning will improve my course grades
The advantages of digital learning outweigh the disadvantages
Overall, digital learning is advantageous
My lecturers’ instructions on how to use the digital technologies for learning are difficult to follow
It is difficult to learn how to use digital technologies for learning
It is easy to operate digital technologies for learning
I find digital learning to be enjoyable
The actual process of digital learning is pleasant
I have fun during digital learning
I think that digital learning should replace face-to-face learning in the long term
I welcome the increasing relocation of educational processes to virtual space, i.e., face-to-face teaching being replaced with digital teaching
I am confident that digital teaching content can be taught without major obstacles
I intend to use digital technologies for learning regularly in the future
I intend to use digital technologies in the future when preparing projects, papers, and assignments
I intend to use digital technologies for learning frequently in the future

10,

**Table 11:** Survey questions: Digital competences

*Digital competences* The following questions relate to your personal use of media and technologies in digital environments. This includes using digital media in your private life but also for work or study related purposes Please rate the following statements as honestly and sincerely as possible
I can identify and use appropriate sources in digital environments based on my information needs
I can use my search strategies in digital environments
I am critical about information, sources, and data in digital environments
I can store digital information and data securely
I can retrieve the information that I have stored
I can retrieve information that I have stored from different environments
I can communicate using different digital media
I can cite information and files from digital environments
I can edit files and documents collaboratively with others using digital media
I can apply behavioral rules in digital interactions and collaborations
I can actively participate in society using digital media
I can share my experiences with digital media in interactions with others
I can use familiar apps and programs according to my needs
I can design my digital products in various formats
I can edit and merge digital content in different formats
I can present digital content in different formats
I know about the dangers and risks in digital environments and consider them
I can protect my privacy in digital environments through appropriate measures
I can regularly update my security settings
I can use digital technologies in a healthy and environmentally responsible way
I can use digital tools and platforms according to my needs
I can adapt digital tools for personal use
I can independently use digital learning opportunities and appropriate tools
I can organize digital learning resources independently
I can develop solutions for technical problems
I know about the functioning and basic principles of digital systems
I identify algorithmic structures in the tools I use
I can analyze the effect of media in digital environments
I can evaluate interest-driven dissemination and the dominance of topics in digital space
I can reflect on the opportunities and risks of media use for my own media use
I can analyze the benefits of learning activities and services in digital environments
I can analyze the risks of learning activities and services in the digital space

11,

**Table 12:** Survey questions: Independent learning abilities

*Independence of learning* The following questions relate to your personal learning experiences in the context of your studies in general Please rate the following statements as honestly and sincerely as possible
I enjoy finding information about new topics on my own
Even when tasks are difficult, I try to stick with them
I am open to new ways of doing familiar things
I enjoy being set a challenge
I tend to be motivated to work by assessment deadlines
I take responsibility for my learning experiences
I enjoy learning experiences

12,

**Table 13:** Survey questions: Self-organization abilities

*Self-organization* The following questions relate to your personal learning experiences in general, regardless of whether you learn digitally or in presence at the university Please rate the following statements as honestly and sincerely as possible
In my studies, I am self-disciplined and I find it easy to set aside reading and homework time
I am able to manage my study time effectively and complete assignments on time
In my studies, I set goals and have a high degree of initiative
When it comes to learning and studying, I am a self-directed person
I plan out my week’s work in advance, either on paper or in my head
To make sure you are paying attention, please click “agree” here
The following questions relate to your personal learning experiences in relation to digital-only learning. The focus here is on how you learn when you cannot physically learn or attend events at the university Please rate the following statements as honestly and sincerely as possible
Not being at the university campus hinders me from studying
I am not able to organize my time during digital learning effectively
I lack the daily routine due to absence of classes at university
I find it difficult to get up in the morning without having a scheduled class
I manage to complete the assignments for online courses
I am more systematic and organized during digital learning

13 and

**Table 14:** Survey questions: User resistance

*Change from face-to-face to digital learning* The following questions relate to your personal view on the change from face-to-face to digital learning Please rate the following statements as honestly and sincerely as possible
I will not comply with the change from face-to-face to digital learning
I will not cooperate with the change from face-to-face to digital learning
I oppose the change from face-to-face to digital learning
I do not agree with the change from face-to-face to digital learning

14.

### Appendix B: Exploratory factor analysis and Cronbach’s alpha

See Tables 15, 16 and 17.

**Table 15:** EFA and CA: TAM

Factor/construct	Explained variance (%)	Factor loading	CA	Measurement items	Justification for deleting item after EFA
1: Attitude toward using	19	0.867	0.800	I think that digital learning should replace face-to-face learning in the long term	*No items had to be deleted after EFA*
		0.841		I welcome the increasing relocation of educational processes to virtual space, i.e., face-to-face teaching being replaced with digital teaching	
		0.634		I am confident that digital teaching content can be taught without major obstacles	
2: Perceived usefulness	18	0.790	0.817	Digital learning will improve my course grades	
		0.752		The advantages of digital learning outweigh the disadvantages	
		0.801		Overall, digital learning is advantageous	
3: Perceived ease of use	18	0.795	0.749	My lecturers’ instructions on how to use the digital technologies for learning are difficult to follow	
		0.844		It is difficult to learn how to use digital technologies for learning	
		0.720		It is easy to operate digital technologies for learning	
4: Behavioral intention to use	17	0.770	0.807	I intend to use digital technologies for learning regularly in the future	
		0.713		I intend to use digital technologies in the future when preparing projects, papers, and assignments	
		0.837		I intend to use digital technologies for learning frequently in the future

**Table 16:** EFA and CA: Digital competences

Factor/construct	Explained variance	Factor loading	CA	Measurement items	Justification for deleting item after EFA
1: Problem-solving I	12	0.671	0.779	I can use digital tools and platforms according to my needs	
		0.585		I can adapt digital tools for personal use	
		0.654		I can independently use digital learning opportunities and appropriate tools	
		0.609		I can organize digital learning resources independently	
2: Analyzing and reflecting	11	0.686	0.835	I can analyze the effect of media in digital environments	
		0.684		I can evaluate interest-driven dissemination and the dominance of topics in digital space	
		0.690		I can reflect on the opportunities and risks of media use for my own media use	
		0.750		I can analyze the benefits of learning activities and services in digital environments	
		0.758		I can analyze the risks of learning activities and services in the digital space	
3: Safety and security	8	0.516	0.747	I know about the dangers and risks in digital environments and consider them	
		0.763		I can protect my privacy in digital environments through appropriate measures	
		0.701		I can regularly update my security settings	
		0.759		I can use digital technologies in a healthy and environmentally responsible way	
4: Information and data literacy	8	*0.457*	0.721	*I can identify and use appropriate sources in digital environments based on my information needs*	*Item refers more to the correct use of the information sources and corresponding tools and thus stands out from the other items*
		0.610		I can use my search strategies in digital environments	
		0.644		I am critical about information, sources, and data in digital environments	
		*0.409*		*I can store digital information and data securely*	*Item also refers to data security and thus cannot be clearly distinguished from factor 3*
		0.670		I can retrieve the information that I have stored	
		0.670		I can retrieve information that I have stored from different environments	
5: Problem-solving II	8	0.799	0.812	I can develop solutions for technical problems	
		0.762		I know about the functioning and basic principles of digital systems	
		0.776		I identify algorithmic structures in the tools I use	
6: Digital content creation	7	*0.130*	0.794	*I can use familiar apps and programs according to my needs*	*Item does not relate to specific actions in connection to digital content or products but rather broadly relates to the use of apps and programs*
		0.793		I can design my digital products in various formats	
		0.816		I can edit and merge digital content in different formats	
		0.670		I can present digital content in different formats	
7: Communication and collaboration	7	*0.372*	0.740	*I can communicate using different digital media*	*Deleted four items target specific and different tasks within this competence area, while the two remaining items are broader and less precisely formulated, and therefore cover this competence area better*
		*0.123*		*I can cite information and files from digital environments*	
		*0.378*		*I can edit files and documents collaboratively with others using digital media*	
		*0.432*		*I can apply behavioral rules in digital interactions and collaborations*	
		0.791		I can actively participate in society using digital media	
		0.759		I can share my experiences with digital media in interactions with others	

The italic emphasis highlights the items that were deleted after EFA

**Table 17:** EFA and CA: General self-organization abilities, Self-organization abilities during digital learning, Independent learning abilities, User resistance

Factor/construct	Explained variance (%)	Factor loading	CA	Measurement items	Justification for deleting item after EFA
1: General self-organization abilities	17	0.772	0.845	In my studies, I am self-disciplined and I find it easy to set aside reading and homework time	
		0.783		I am able to manage my study time effectively and complete assignments on time	
		0.774		In my studies, I set goals and have a high degree of initiative	
		0.726		When it comes to learning and studying, I am a self-directed person	
		0.658		I plan out my week’s work in advance, either on paper or in my head	
2: Self-organization abilities during digital learning	15	0.726	0.849	Not being at the university campus hinders me from studying	
		0.701		I am not able to organize my time during digital learning effectively	
		0.847		I lack the daily routine due to absence of classes at university	
		0.734		I find it difficult to get up in the morning without having a scheduled class	
		*0.250*		*I manage to complete the assignments for online courses*	*Unlink the other items that measure typical characteristics of self-organization abilities, like e.g., routines, time management, or motivation, this item relates to completing assignments, but without a direct link to self-organization abilities*
		0.653		I am more systematic and organized during digital learning	
3: User resistance	13	0.780	0.844	I will not comply with the change from face-to-face to digital learning	
		0.857		I will not cooperate with the change from face-to-face to digital learning	
		0.753		I oppose the change from face-to-face to digital learning	
		0.734		I do not agree with the change from face-to-face to digital learning	
4: Independent learning abilities	11	0.640	0.682	I enjoy finding information about new topics on my own	
		0.614		Even when tasks are difficult, I try to stick with them	
		0.670		I am open to new ways of doing familiar things	
		0.681		I enjoy being set a challenge	
		− *0.239*		*I tend to be motivated to work by assessment deadlines*	*Item is the only negatively worded item on this scale and is closely related to factor 1 since it is closely related to time management*
		*0.398*		*I take responsibility for my learning experiences*	*Item is very abstract, especially compared to the other items in this scale*
		0.540		I enjoy new learning experiences	

The italic emphasis highlights the items that were deleted after EFA

### Appendix C: Initial and adjusted list of hypotheses

See Table

**Table 18:** Initial and adjusted list of hypotheses

Initial hypotheses	Adjusted hypotheses after EFA
H1a: Information and data literacy competences of students have a positive influence on their perceived ease of using digital technologies for learning	H1a: Information and data literacy competences of students have a positive influence on their perceived ease of using digital technologies for learning
H1b: Communication and collaboration competences of students have a positive influence on their perceived ease of using digital technologies for learning	H1b: Communication and collaboration competences of students have a positive influence on their perceived ease of using digital technologies for learning
H1c: Digital content creation competences of students have a positive influence on their perceived ease of using digital technologies for learning	H1c: Digital content creation competences of students have a positive influence on their perceived ease of using digital technologies for learning
H1d: Safety and security competences of students have a positive influence on their perceived ease of using digital technologies for learning	H1d: Safety and security competences of students have a positive influence on their perceived ease of using digital technologies for learning
H1e: Problem-solving competences of students have a positive influence on their perceived ease of using digital technologies for learning	H1e: Problem-solving I competences of students have a positive influence on their perceived ease of using digital technologies for learning
	H1f: Problem-solving II competences of students have a positive influence on their perceived ease of using digital technologies for learning
H1f: Analyzing and reflecting competences of students have a positive influence on their perceived ease of using digital technologies for learning	H1g: Analyzing and reflecting competences of students have a positive influence on their perceived ease of using digital technologies for learning
H2a: General self-organization abilities have a positive influence on the perceived usefulness of digital learning	H2a: General self-organization abilities have a positive influence on the perceived usefulness of digital learning
H2b: Self-organization abilities during digital learning have a positive influence on the perceived usefulness of digital learning	H2b: Self-organization abilities during digital learning have a positive influence on the perceived usefulness of digital learning
H3a: General self-organization abilities have a positive influence on the attitude toward digital learning	H3a: General self-organization abilities have a positive influence on the attitude toward digital learning
H3b: Self-organization abilities during digital learning have a positive influence on the attitude toward digital learning	H3b: Self-organization abilities during digital learning have a positive influence on the attitude toward digital learning
H4: Independent learning abilities have a positive influence on the perceived usefulness of digital learning	H4: Independent learning abilities have a positive influence on the perceived usefulness of digital learning
H5: Independent learning abilities have a positive influence on the attitude toward digital learning	H5: Independent learning abilities have a positive influence on the attitude toward digital learning
H6: The attitude toward digital learning has a negative influence on the resistance to digital learning	H6: The attitude toward digital learning has a negative influence on the resistance to digital learning
H7: The perceived ease of using digital technologies for learning has a positive influence on the perceived usefulness of digital learning	H7: The perceived ease of using digital technologies for learning has a positive influence on the perceived usefulness of digital learning
H8: The perceived ease of using digital technologies for learning has a positive influence on the attitude toward digital learning	H8: The perceived ease of using digital technologies for learning has a positive influence on the attitude toward digital learning
H9: The perceived usefulness of digital learning has a positive influence on the attitude toward digital learning	H9: The perceived usefulness of digital learning has a positive influence on the attitude toward digital learning
H10: The perceived usefulness of digital learning has a positive influence on the behavioral intention to use digital technologies for learning	H10: The perceived usefulness of digital learning has a positive influence on the behavioral intention to use digital technologies for learning
H11: The attitude toward digital learning has a positive influence on the behavioral intention to use digital technologies for learning	H11: The attitude toward digital learning has a positive influence on the behavioral intention to use digital technologies for learning

18.
